# Changes in growth performance, immune function, and meat quality of yellow-feathered broilers fed *Scarabaeiform larvae* meal during early-mid growth phases

**DOI:** 10.3389/fvets.2025.1638495

**Published:** 2025-08-08

**Authors:** Shuaihu Chen, Tingting Liu, Hong Shen, Jungang Wang

**Affiliations:** ^1^College of Animal Science and Technology, Shihezi University, Shihezi, China; ^2^College of Agriculture, Shihezi University, Shihezi, China

**Keywords:** protein feed, yellow-feathered broilers, *Scarabaeiform larva*, meat quality, phased feeding

## Abstract

Amid the global food security crisis, protein feed shortages—particularly soybean meal—severely constrain sustainable development in the livestock industry. *Scarabaeiform larvae* (Sl) emerge as a highly promising alternative protein source due to their nutritional and biological properties, yet their dose-stage dynamics in poultry feeding systems require systematic validation. This study evaluated replacing soybean meal with Sl meal at different doses and stages on growth performance, slaughter traits, meat quality, immune function, and gut microbiota in yellow-feathered broilers. A total of 150 one-day-old male broilers were randomly allocated into five groups: C (control, 0% Sl throughout), 4% Sl (Full) (4% Sl throughout), 8% Sl (Full) (8% Sl throughout), 4% Sl (Phased) (4% Sl from day 1–42 then 0%), and 8% Sl (Phased) (8% Sl meal from day 1–42 then 0%). The trial spanned three phases (1–21, 22–42, and 43–63 days), with slaughter sampling on day 63. During 1–42 days, the 4% Sl meal groups (4% Sl (Full), 4% Sl (Phased) groups) exhibited higher body weight and weight gain compared to controls (*p* < 0.05). From 43 to 63 days, 4% Sl (Phased) and 8% Sl (Phased) groups (supplemented with Sl in early-mid phases) achieved better growth performance, whereas continuous Sl supplementation (4% Sl (Full), 8% Sl (Full) groups) reduced growth rates (*p* < 0.05). 4% Sl (Phased) and 8% Sl (Phased) groups showed increased abdominal lipid deposition, elevated intramuscular fat (*p* < 0.05), and higher levels of multiple fatty acids. Immunologically, full-term Sl groups (4% Sl (Full), 8% Sl (Full) groups) had significantly higher pro-inflammatory cytokine levels (IFN-γ, IL-1β, TNF-α) than the control (*p* < 0.05), while 4% Sl (Phased) and 8% Sl (Phased) groups reduced this immune response (*p* < 0.05), and 4% Sl (Phased) group additionally increased serum IgA and IgG levels (*p* < 0.05). Significant differences in gut microbiota community structure were observed among groups (*p* < 0.05), with correlation analysis indicating that 4% Sl (Phased) group-enriched *Faecalibacterium* was associated with enhanced lipid deposition and regulation of antioxidant and inflammatory cytokine levels (*p* < 0.05). These findings demonstrate that phased 4% Sl supplementation (1–42 days) improves growth performance, intramuscular lipid deposition, and humoral immunity while mitigating excessive immune activation caused by prolonged Sl use. This strategy effectively replaces partial soybean meal in early-mid growth phases, providing a theoretical basis for the application of Sl meal in poultry feed.

## 1 Introduction

Global food security remains a critical challenge, with approximately 790 million people worldwide suffering from hunger due to insufficient supply in global food systems (1). This issue directly impacts feed security, as competition between food and feed resources poses risks to feed availability (2). The intensive and large-scale development of the livestock industry has exacerbated feed resource shortages, making stable feed supply-a foundational requirement for animal husbandry-increasingly urgent. The connection between food security and feed demand constrains the sustainable development of poultry farming. Among the various grain-derived feed resources, the shortage of protein feed, particularly soybean meal resources, has become the most severe constraining factor. Therefore, the exploration of sustainable protein alternatives has emerged as a research priority (3), prompting the development of diverse feed protein sources, However, current mainstream alternative protein sources face significant challenges: plant proteins (such as rapeseed meal, cottonseed meal, etc.) are often limited by anti-nutritional factors and amino acid imbalances (4), and their production intensifies competition with human food crops for land resources; microbial single-cell protein, while high in protein content, suffers from high production costs and complex nucleic acid removal processes (5); traditional fishmeal faces resource depletion and price volatility due to overfishing (6), significantly driving up farming costs. These limitations highlight the urgent need to develop novel protein sources that are more resource-efficient, environmentally friendly, and economically viable, among which insects demonstrate unique advantages. As one of the earliest life forms, insects exhibit remarkable biodiversity, currently comprising 75% of all species in the animal kingdom (7), and are globally distributed. Owing to their rich nutritional composition-including high-quality protein and lipids and the growing demand for livestock products (8), research and application of insect-based feeds have advanced significantly (9–11).

*Scarabaeiform larva* (Sl) offer unique advantages: they are globally distributed and nutritionally dense, even consumed as edible insects in some regions, such as South Korea (12). On a dry matter basis, Sl contain over 40% crude protein-comparable to premium soybean meal and approximately 13% fat rich in essential fatty acids (e.g., linoleic acid) and functional fatty acids (e.g., oleic acid) (13). Beyond nutritional value, Sl exhibit pharmacological potential: studies demonstrate their antioxidant and anti-inflammatory properties, such as Sl derived peptide extracts mitigating oxidative stress via the NRF2-ARE signaling pathway (14), and attenuating carbon tetrachloride (CCl_4_)- and β-D-galactosamine-induced acute liver injury in rats by reducing serum alanine transaminase (ALT) and aspartate transaminase (AST) activity (15). Furthermore, Sl efficiently convert agricultural organic waste (e.g., manure, crop residues) into nutritional resources (16), thereby not only reducing environmental pollution and resource depletion associated with conventional waste management but also yielding nutrient-dense insect biomass, highlighting their cost efficiency in resource recycling.

Previous studies demonstrate that: a 36% inclusion of black soldier fly (*Hermetia illucens*) larvae meal in pikeperch (*Sander lucioperca*) diets reduced final body weight by 7%, while lower doses improved growth performance (17); similarly, a 20% replacement of fish meal with black soldier fly meal in the formulated diets for largemouth bass (*Micropterus salmoides*) effectively sustained growth rates, whereas a 40% replacement resulted in significant adverse effects (18); high-dose silkworm pupae meal supplementation in Rhode Island Red × Fayoumi crossbred chickens diets enhanced weight gain during days 1–42 but reduced performance after day 42 (19); complete replacement of soybean meal with black soldier fly meal in piglet diets showed no impact during days 1–14 but significantly decreased weight gain at days 15–28 (20). These findings collectively confirm that insect-based feed ingredients exhibit dose- and stage-dependent effects on efficacy (–18 to 20), which may be attributed to the accumulation of certain elements (21).

Currently, the utilization of Sl as poultry feed remains unreported, and their safety and nutritional efficacy lack empirical validation. To address this gap while leveraging the dose-stage dynamics observed in other insect proteins, this study targeted the 42–63 days period-the growth peak phase in fast-growing yellow-feathered broilers-as the Sl meal withdrawal phase, systematically comparing full-term (1–63 days) versus phased (1–42 days) supplementation regimes. The findings aim to provide foundational data and theoretical support for the integration of Sl-based ingredients into sustainable poultry feed systems.

## 2 Materials and methods

### 2.1 Animals and ethics statement

All broilers are raised and euthanized in strict accordance with the guidelines of the Animal Experiment Ethics Committee of the College of Animal Science and Technology of Shihezi University, approval number: 2023-031.

### 2.2 Experimental design and broilers management

This experiment utilized a single-factor completely randomized design involving 150 healthy 1-day-old male fast-growing yellow-feathered broilers, randomly divided into 5 treatment groups (6 replicates/group, 5 birds/replicate). Dietary treatments were structured as follows: Control (C) received Sl0 basal diet (0% Sl meal substitution) throughout the 63-day trial; 4% Sl (Full) and 8% Sl (Full) were fed Sl4 (4% Sl meal isoproteic substitution for soybean meal) and Sl8 (8% substitution) diets, respectively, for the entire duration; 4% Sl (Phased) and 8% Sl (Phased) underwent phased feeding, with Sl4 and Sl8 diets administered during days 1–42, followed by a switch to Sl0 from days 43–63 [The dose levels (4 and 8%) (22–24) and phased regimen (42-day threshold) (19–25) were determined based on feed application studies of insect proteins such as yellow mealworm and black soldier fly]. All diets were isonitrogenous and isocaloric. Birds were housed in thermostatically controlled 2 × 3 vertical cages (2 m × 0.9 m × 1.8 m), with temperature regulated from 32 to 35°C on day 1 to a stabilized 22°C during the final 2 weeks. Ad libitum feed/water access was maintained, while immunization and disinfection protocols strictly followed farm standards. On day 63, 6 birds per group (1/replicate) were slaughtered for sampling to evaluate growth performance, slaughter traits, meat quality, and intestinal microbiota. All procedures adhered to consistent environmental controls and analytical methodologies to ensure data comparability.

### 2.3 Diets and analyses

The nutritional profiles of Sl meal are presented in Table 1, while the composition and nutritional profiles of the experimental diets are shown in Table 2. Sl meal refers to the 2nd-instar larvae of Protaetia brevitarsis (white star flower chafer) raised with bran that were oven-dried, ground into powder, sieved through a 60-mesh sieve, and stored for use; all required material for the experiment was prepared at once and thoroughly homogenized to minimize batch variations. The basal diet was formulated to meet the Nutrient Requirements for Yellow Feather Broilers (NY/T 3645-2020) (25). The nutritional components of the Sl meal were determined using modified methods based on previous literature (26), whereas corn and soybean meal components were analyzed by Near-Infrared Spectroscopy (NIRS) using a DS2500 (F) analyzer (FOSS A/S, Denmark) with FOSS Check Sample calibration.

**Table 1:** Composition analysis of SL meal (dry matter basis).

Item	Content
Nutrient levels	
Moisture, %	68
Crude protein (CP), %	43.21
Ether extract (EE), %	13.35
Neutral detergent fiber (NDF), %	7.45
Crude ash, %	28.53
Nitrogen-free extract, %	7.45
ME, MJ/kg	13.20
Lysine, %	2.42
Methionine, %	1.22
Calcium, %	0.22
Total phosphorus, %	0.43
Fatty acid profile	
SFAs, %	
Myristic (C14:0)	1.54
Pentadecanoic (C15:0)	1.27
Palmitic (C16:0)	4.29
Heptadecanoic (C17:0)	2.01
Stearic (C18:0)	3.97
Arachidic (C20:0)	5.24
Heneicosanoic (C21:0)	3.34
Behenic (C22:0)	6.15
Tricosanoic (C23:0)	6.89
Lignoceric (C24:0)	5.83
MUFAs, %	
Myristoleic (C14:1)	1.38
Pentadecenoic (C15:1)	1.59
Palmitoleic (C16:1)	3.50
Heptadecenoic (C17:1)	2.38
Oleic (C18:1)	8.96
Gadoleic (C20:1)	3.29
PUFAs, %	
Linoleic (C18:2)	6.31
Alpha-Linolenic (C18:3)	3.18
Gamma-Linolenic (C18:3)	3.02
Eicosadienoic (C20:2)	3.39
Dihomo-γ-linolenic (C20:3)	6.94
Arachidonic (C20:4)	3.29
Eicosapentaenoic (C20:5)	3.39
Docosadienoic (C22:2)	3.29
Docosahexaenoic (C22:6)	2.91

Metabolizable energy (ME) was a calculated value, while the remaining components were measured values. Proximate nutrients (moisture, crude ash, crude protein, ether extract), calcium, and phosphorus were determined by conventional chemical analysis; lysine and methionine were measured by liquid chromatography; and fatty acid profiles were analyzed by gas chromatography–mass spectrometry (GC–MS).

**Table 2:** Composition and nutrient levels of basal diets (%, dry matter basis).

Item	Starter (d 1–21)			Grower (d 22–42)			Finisher (d 43–63)		
	Sl0^2^	Sl4^2^	Sl8^2^	Sl0^2^	Sl4^2^	Sl8^2^	Sl0^2^	Sl4^2^	Sl8^2^
Ingredients, %									
Maize	40.85	42.32	43.71	46.83	48.22	49.64	50.50	52.00	53.42
Soybean meal	43.35	38.90	34.48	37.34	32.93	28.50	33.38	28.90	24.53
Sl meal	0.00	4.00	8.00	0.00	4.00	8.00	0.00	4.00	8.00
Soybean oil	9.05	8.05	7.10	9.35	8.36	7.37	10.00	9.00	8.00
Premix^1^	5.00	5.00	5.00	5.00	5.00	5.00	5.00	5.00	5.00
L-Lysine HCl	0.10	0.11	0.13	0.10	0.11	0.12	0.00	0.00	0.00
DL-Methionine	0.20	0.17	0.14	0.20	0.17	0.16	0.14	0.12	0.08
Calcium carbonate	0.30	0.30	0.26	0.26	0.26	0.26	0.20	0.20	0.18
Calcium hydrogen phosphate	1.15	1.15	1.18	0.92	0.95	0.95	0.78	0.78	0.79
Total	100	100	100	100	100	100	100	100	100
Nutrient levels^3^									
Crude protein, %	21.51	21.50	21.51	19.50	19.50	19.50	17.99	18.00	18.02
ME, MJ/kg	12.38	12.38	12.39	12.81	12.81	12.81	13.19	13.20	13.20
Lysine, %	1.29	1.29	1.29	1.15	1.15	1.15	0.97	0.96	0.98
Methionine, %	0.50	0.50	0.50	0.48	0.49	0.48	0.41	0.40	0.40
Calcium, %	1.02	1.01	1.00	0.92	0.92	0.92	0.84	0.83	0.84
Total phosphorus, %	0.74	0.74	0.74	0.67	0.67	0.67	0.62	0.62	0.62

1 Supplying per kilogram feed: 10,000 IU vitamin A, 10,000 IU vitamin D3, 4 mg vitamin E, 0.75 mg vitamin K, 0.7 mg thiamine, 1.8 mg riboflavin, 0.55 mg pyridoxine, 0.01 mg vitamin B12, 11.25 mg niacin, 3.5 mg pantothenic acid, 0.75 mg folic acid, 0.05 mg biotin, 5 g NaCl, 4.25 g Ca, 1.125 g P, 75 mg Zn, 100 mg Mn, 125 mg Fe, 15 mg Cu, 0.75 mg I, 0.3 mg Se, 0.25 g Lysine.

2 SL0: Control diet without supplementation of Sl meal; SL4: Diet containing 4% Sl meal as an isoproteic substitution for soybean meal; SL8: Diet containing 8% Sl meal as an isoproteic substitution for soybean meal.

3 ME values were calculated from data provided by NY/T 3645-2020, while the others were measured values.

### 2.4 Growth performance

Initial body weight was measured before the chicks were housed, followed by daily recording of mortality and culling numbers along with body weight measurements. At 21, 42, and 63 days of age, birds were fasted for 12 h prior to weight measurement. Feed intake was accurately recorded on a replicate group basis. Based on the collected data, average weight gain (AWG) and feed conversion ratio (FCR: total feed consumed during the production cycle divided by total weight gain) were calculated for the starter (0–21 days), grower (22–42 days), and finisher (43–63 days) periods, respectively. AWG was determined by dividing the weight gain of individual birds by the duration of each corresponding growth phase.

### 2.5 Slaughter performance

At day 63, one bird with comparable average body weight (BW) was selected from each replicate and euthanized via cervical exsanguination. Following defeathering, slaughter performance metrics-including slaughter rate, half-eviscerated rate, fully eviscerated rate, breast muscle yield, thigh muscle yield, and abdominal fat percentage-were determined according to “Agricultural Industry Standard of China (NY/T 823-2020)” for poultry production performance evaluation (27).

### 2.6 Meat quality

Immediately after the yellow-feathered broilers were slaughtered, the meat color of the same muscle was measured using an NR110 high-quality spectrophotometer (3NH Technology Co., Ltd, Shenzhen, China) with D65 illuminant, 8 mm diameter aperture size, 10° standard observer angle, and transverse to fiber orientation. The color measurements were carried out according to redness (a*), yellowness (b*), and lightness (L*), following the previous method (28). Select approximately 100 g of pectoral muscle samples, homogenize them, and analyze the meat composition using the Series3000 Near-Infrared Meat Quality Analyzer (NextInstruments, Australia). The left pectoralis muscle sample was heated in an 80°C water bath until reaching a core temperature of 70°C. Cooking loss was expressed as the percentage of weight reduction before and after heating. A C-LM 3B texture analyzer (Beijing Tianxiang Feiyu Instrument Co., Ltd., Beijing, China) was employed to measure Warner-Bratzler shear force (expressed in kgf) using six cylindrical muscle samples (1.27 cm diameter) aligned parallel to muscle fiber orientation. Shear force was calculated as kg/cm^2^ for each columnar muscle sample. For drip loss determination: The left pectoralis muscle was sectioned into 2 cm × 3 cm × 5 cm blocks. These meat blocks were suspended in 4°C refrigeration for 24 h. The initial weight was recorded as W1 and the post-storage weight as W2. Drip loss percentage was calculated using the formula: (W1 − W2)/W1 × 100%.

### 2.7 Fatty acids in pectoralis major muscle

Fatty acid analysis was conducted using left pectoral muscle samples from broiler chickens. Aliquot samples were freeze-dried for 48 h in an LGJ-10 freeze dryer (Beijing Songyuan Huaxing Technology Development Co., Ltd., operating at −60°C and 20 Pa), followed by pulverization and refrigerated storage.

For total lipid extraction, 0.4 g of pulverized sample was mixed with 4 mL sodium methoxide (20 g/L), vortexed for 5 min, incubated at 50°C for 15 min, then cooled. After adding 4 mL 2% sulfuric acid-methanol solution (2 mL/100 mL), the mixture was vortexed for 5 min, incubated at 50°C for 1 h, cooled, and centrifuged at 3,500 rpm for 5 min. The upper organic layer was transferred to a tube containing 0.25 g activated carbon and 0.3 g anhydrous sodium sulfate, gently shaken until decolorized, and left standing for 1 h. Following centrifugation (3,500 rpm, 5 min), the organic layer was transferred, centrifuged at 13,000 rpm for 5 min, filtered through a 0.45 μm PTFE membrane, and stored at 4°C in GC vials.

Analysis was performed using an Agilent 8890-7000 D GC–MS with FID detector, BPX70 capillary column (30 m × 0.32 mm ID, 0.25 μm film), helium carrier gas (1 mL/min), 250°C injector/detector, and a 50:1 split ratio. The temperature program initiated at 80°C (3 min hold), ramped at 6°C/min to 195°C (2 min hold), then 1°C/min to 230°C (total 87 min).

Fatty acid methyl esters were identified using 37-component FAME standards (AccuStandard) via retention time matching, with data processed by ChromQuest 5.0 software (v3.2.1).

### 2.8 Antioxidant capacity in the pectoralis major muscle

Left pectoral muscle samples were collected from slaughtered yellow-feathered broilers, immediately frozen in liquid nitrogen, and stored in a −80°C ultra-low temperature freezer for subsequent antioxidant analysis. The colorimetric method was used to measure malondialdehyde (MDA), superoxide dismutase (SOD), catalase (CAT), and glutathione peroxidase (GSH-Px) levels in the pectoral muscle, following the manufacturer’s instructions of the assay kits (Shanghai Enzyme-linked Biotechnology Co., Ltd., China).

### 2.9 Plasma immune and antioxidant biomarker analysis

On day 63 of the experiment, six yellow-feathered broilers were randomly selected from each group for blood collection. Following a 12-h fasting period, approximately 3 mL of cardiac blood was collected from each bird into tubes. The samples were allowed to clot at room temperature for 40 min, followed by centrifugation at 3,500 r/min for 20 min at 4°C. The resulting plasma supernatant was stored at −20°C for subsequent analysis. Plasma immunoglobulin levels (IgA, IgG, IgM) and cytokine concentrations (IL-1β, IL-6, IFN-γ, TNF-α) were determined using enzyme-linked immunosorbent assay (ELISA), while oxidative stress markers including malondialdehyde (MDA), superoxide dismutase (SOD), catalase (CAT), and glutathione peroxidase (GSH-Px) were quantified through colorimetric methods. All analytical procedures were performed in strict accordance with the manufacturer’s protocols provided in commercial kits (Shanghai Enzyme-linked Biotechnology Co., Ltd., China).

### 2.10 Cecal microbial diversity

Given the critical linkage between gut microbial diversity following dietary interventions and host intestinal health (29), cecal microbiota composition was profiled using high-throughput sequencing technology. The cecal microbial diversity was investigated using cecal content samples collected during slaughter. Bacterial genomic DNA was extracted from the cecal contents employing a magnetic bead-based method with the TIANGEN DNA extraction kit (TIANGEN Biotech Co., Ltd.). Subsequent experimental procedures, including PCR amplification, library preparation, and high-throughput sequencing, were conducted by Novogene Co., Ltd. (Beijing). Amplification targeted the hypervariable V4 region of the 16S rRNA gene using primer pairs 515F and 806R, followed by library construction with the NEB Next^®^ Ultra™ II FS DNA Library Prep Kit. Paired-end sequencing (2 × 250 bp) was performed on the Illumina NovaSeq 6000 platform.

Bioinformatic processing, executed through QIIME2 pipelines, included: (1) raw data quality control via fastp, (2) paired-end read merging using FLASH to generate Raw Tags, (3) denoising and Amplicon Sequence Variant (ASV) identification via DADA2, and (4) taxonomic annotation against the SILVA SSU Ref NR 99 database (release 138.1 at 99% similarity). Following denoising and ASV calling with DADA2, the average number of high-quality sequences per sample was 67,825 reads (range: 34,346–95,824 reads). To ensure even sampling depth for subsequent alpha and beta diversity analyses, all samples were rarefied to a depth of 34,000 reads per sample prior to calculating diversity metrics. Down-stream analyses performed on the Novogene Cloud Platform encompassed alpha diversity indices (Shannon, Chao1, Simpson), beta diversity metrics, and differential taxa identification through Linear Discriminant Analysis Effect Size (LEfSe). All an-alytical workflows adhered to standardized QIIME2 protocols for microbiome characterization.

### 2.11 Statistical analysis

All statistical analyses in this study were processed with the SPSS 26.0 statistical software. Data were analyzed by 1-way analysis of variance (ANOVA) procedure and differences were examined using Tukey’s multiple range test. Data were presented as mean with their pool standard error of the mean (SEM) and statistical significance was defined as a *p* value < 0.05.

## 3 Results

### 3.1 Growth performance

Table 3 shows that body weight: At 21 and 42 days of age, the 4% Sl (Full) group showed significantly higher body weights than the C, 8% Sl (Full) and 8% Sl (Phased) groups (*p* < 0.05). The 4% Sl (Phased) group demonstrated significantly superior body weight compared to other groups at 63 days (*p* < 0.05), while the 8% Sl (Full) group was significantly lower than all other groups (*p* < 0.05).

**Table 3:** Impact of Sl meal on growth performance of broiler chickens.

Item	C	4% Sl (Full)	8% Sl (Full)	4% Sl (Phased)	8% Sl (Phased)	SEM	*p*-value
Live body weight, g							
1 d	33.94	33.96	34.44	34.18	34.07	0.75	0.307
21 d	422.42^bc^	452.81^a^	423.9^bc^	447.58^ab^	418.81^c^	3.13	<0.001
42 d	1,358.53^b^	1,446.98^a^	1,321.70^b^	1,466.28^a^	1,350.44^b^	7.02	<0.001
63 d	2,339.30^b^	2,315.09^b^	2,184.82^c^	2,463.16^a^	2,302.84^b^	9.32	<0.001
Feed intake per chicken, g							
1–21 d	654.17^b^	697.83^a^	647.17^b^	701.83^a^	637.33^b^	6.04	<0.001
22–42 d	1,985.33^ab^	2,031.50^a^	1,923.17^b^	2,065.00^a^	2,002.00^ab^	13.75	0.002
1–42 d	2,639.50^bc^	2,729.33^ab^	2,570.33^c^	2,766.83^a^	2,639.33^bc^	16.52	<0.001
43–63 d	3,011.50^a^	2,753.33^bc^	2,671.17^c^	3,022.17^a^	2,900.00^ab^	33.36	<0.001
1–63 d	5,651.00^ab^	5,482.67^b^	5,241.50^c^	5,789.00^a^	5,539.33^b^	39.17	<0.001
Body weight gain, g							
1–21 d	388.50^c^	419.00^a^	389.50^bc^	413.33^ab^	384.83^c^	3.60	0.001
22–42 d	936.67^b^	991.67^a^	898.83^b^	1,019.17^a^	931.83^b^	9.69	<0.001
1–42 d	1,326.00^b^	1,410.50^a^	1,288.33^b^	1,432.67^a^	1,316.67^b^	11.53	<0.001
43–63 d	979.83^a^	882.33^b^	862.83^b^	996.17^a^	953.67^a^	12.14	<0.001
1–63 d	2,304.83^b^	2,292.67^b^	2,151.17^c^	2,428.83^a^	2,270.33^b^	17.48	<0.001
Feed conversion ratio, g feed/g weight gain							
1–21 d	1.68	1.67	1.66	1.69	1.66	0.009	0.611
22–42 d	2.12^ab^	2.05^bc^	2.14^a^	2.03^c^	2.15^a^	0.012	0.002
1–42 d	1.99^a^	1.94^b^	2.00^a^	1.93^b^	2.01^a^	0.008	0.004
43–63 d	3.08	3.12	3.10	3.04	3.04	0.013	0.192
1–63 d	2.45^a^	2.39^bc^	2.44^ab^	2.38^c^	2.44^ab^	0.007	0.002

^a,b,c^Values in the same row with no common superscript differ significantly (*p* < 0.05).

Feed Intake: The 4% Sl (Full) and 4% Sl (Phased) groups exhibited significantly higher feed intake than other groups during 1–21 days and 1–42 days (*p* < 0.05), with no significant difference from the C group during 22–42 days (*p* > 0.05). The 8% Sl (Full) group showed significantly lower feed intake than other groups throughout 1–63 days, with no significant difference from the C group during 1–42 days (*p* > 0.05), but significantly lower than C, 4% Sl (Phased) and 8% Sl (Phased) groups during 43–63 days (*p* < 0.05).

Weight Gain: The 4% Sl (Full) had significantly higher weight gain than C, 8% Sl (Full) and 8% Sl (Phased) groups during both 1–21 days and 22–42 days. From 1 to 42 days, both 4% Sl (Full) and 4% Sl (Phased) groups showed significantly higher weight gain than C, 8% Sl (Full) and 8% Sl (Phased) groups. During 42–63 days, 4% Sl (Phased) group achieved significantly higher weight gain than other groups, while 4% Sl (Full) and 8% Sl (Full) groups were significantly lower than others (*p* < 0.05). Throughout the entire period, 4% Sl (Phased) maintained significantly higher weight gain than other groups, whereas 8% Sl (Full) showed the lowest (*p* < 0.05).

Feed Conversion Ratio (FCR): During 22–42 days, 4% Sl (Full) and 4% Sl (Phased) groups had significantly lower FCR than 8% Sl (Full) and 8% Sl (Phased) groups (*p* < 0.05). From 1 to 42 days, both 4% Sl (Full) and 4% Sl (Phased) groups showed significantly lower FCR than other groups (*p* < 0.05). Throughout 1–63 days, 4% Sl (Phased) group demonstrated significantly lower FCR than C, 8% Sl (Full) and 8% Sl (Phased) groups, while 4% Sl (Full) group had significantly lower FCR than the C group (*p* < 0.05).

### 3.2 Slaughter performance

Table 4 shows that, the abdominal fat ratio in 8% Sl (Phased) group was significantly higher than in both C and 4% Sl (Full) group (*p* < 0.05), while 4% Sl (Phased) group also showed a higher abdominal fat ratio compared to 4% Sl (Full) group (*p* < 0.05). No significant differences were observed among groups for other slaughter performance parameters: slaughter rate, half-chamber rate, full clearance rate, breast muscle yield, or thigh muscle yield (*p* > 0.05).

**Table 4:** Impact of Sl meal on slaughter performance of broiler chickens.

Item	C	4% Sl (Full)	8% Sl (Full)	4% Sl (Phased)	8% Sl (Phased)	SEM	*p*-value
Body weight, g	2,362.00^c^	2,331.67^b^	2,210.33^b^	2,475.17^a^	2,340.50^b^	16.88	<0.001
Slaughter, %	93.30	92.63	93.51	92.92	93.42	0.16	0.395
Half-chamber, %	87.39	87.61	89.70	88.42	87.94	0.38	0.320
Full clearance, %	71.66	72.19	71.82	71.13	72.71	0.22	0.206
Breast muscle, %	10.85	11.56	11.00	10.73	10.07	0.20	0.234
Thigh muscle, %	7.06	6.87	6.99	7.50	7.09	0.11	0.451
Abdominal fat ratio, %	1.67b^c^	1.63^c^	1.90^abc^	2.05^ab^	2.20^a^	0.06	0.001

^a,b,c^Values in the same row with no common superscript differ significantly (*p* < 0.05).

### 3.3 Meat quality

Table 5 shows that, the 4% Sl (Full) and 4% Sl (Phased) groups had significantly higher pectoral muscle fat percentages compared to the C and 8% Sl (Full) groups (*p* < 0.05); The C group exhibited significantly higher L* values (lightness) than all other groups. In contrast, the 4% Sl (Phased) group showed the lowest a* values (redness), which were significantly lower than those of the 4% Sl (Full) group (*p* < 0.05); No significant differences were observed among the groups in shear force, water-holding capacity, or cooking loss rate (*p* > 0.05).

**Table 5:** Impact of Sl meal on breast meat quality of broiler chickens.

Item	C	4% Sl (Full)	8% Sl (Full)	4% Sl (Phased)	8% Sl (Phased)	SEM	*p*-value
Composition, %							
Water	71.38	70.66	71.42	70.85	70.80	0.11	0.052
Protein	20.99	20.65	20.98	20.78	20.84	0.04	0.053
Fat	5.68^b^	6.81^ab^	5.88^b^	7.84^a^	7.47^a^	0.22	0.001
Breast color							
L*	42.08^a^	37.81^b^	37.41^b^	35.80^b^	35.36^b^	0.61	0.001
a*	1.36^ab^	1.68^a^	1.06^ab^	0.49^b^	0.63^ab^	0.14	0.034
b*	5.75	6.43	4.96	4.96	4.63	0.25	0.122
Shear force, kgf	21.33	24.16	21.4	20.97	16.10	1.61	0.654
Drip loss percentage, %	0.97	0.98	0.98	0.97	0.97	0.002	0.316
Cooking loss, %	0.84	0.80	0.80	0.79	0.78	0.009	0.385

^a,b,c^Values in the same row with no common superscript differ significantly (*p* < 0.05).

L∗ stands for lightness, a∗ for redness and b∗ for yellowness.

### 3.4 Fatty acids in pectoralis major muscle

#### 3.4.1 Absolute fatty acid content in pectoralis major muscle (g/100 g)

Table 6 shows that, compared to C group: In the 4% Sl (Phased) group, saturated fatty acids (SFAs) including myristic acid, palmitic acid, stearic acid, and tricosanoic acid, as well as polyunsaturated fatty acids (PUFAs) such as linoleic acid, α-linolenic acid, eicosadienoic acid, and dihomo-γ-linolenic acid, along with total SFAs (ΣSFAs), total monounsaturated fatty acids (ΣMUFAs), and total PUFAs (ΣPUFAs), were significantly increased (*p* < 0.05). In the 8% Sl (Phased) group, SFAs (pentadecanoic acid, heptadecanoic acid, arachidic acid, tricosanoic acid), MUFAs (gadoleic acid), and PUFAs (γ-linolenic acid, docosahexaenoic acid) also showed significant increases compared to C group (*p* < 0.05). Additionally, both 4% Sl (Phased) and 8% Sl (Phased) groups exhibited significantly higher levels of omega-3 fatty acids, while the 4% Sl (Phased) group displayed elevated omega-6 fatty acids and a higher ratio of total saturated fatty acids to polyunsaturated fatty acids compared to C group (*p* < 0.05).

**Table 6:** Impact of Sl meal on breast meat fatty acid profile (absolute values).

Item	C	4% Sl (Full)	8% Sl (Full)	4% Sl (Phased)	8% Sl (Phased)	SEM	*p*-value
SFAs, g/100 g							
Myristic (C14:0)	0.27^b^	0.29^b^	0.35^ab^	0.51^a^	0.40^ab^	0.03	0.027
Pentadecanoic (C15:0)	0.12^b^	0.13^ab^	0.13^ab^	0.14^ab^	0.14^a^	0.002	0.038
Palmitic (C16:0)	8.86^b^	10.25^ab^	12.01^ab^	18.52^a^	14.47^ab^	1.13	0.05
Heptadecanoic (C17:0)	0.19^b^	0.20^ab^	0.20^ab^	0.21^ab^	0.22^a^	0.003	0.021
Stearic (C18:0)	2.99^b^	3.67^ab^	4.36^ab^	6.87^a^	5.86^ab^	0.44	0.017
Arachidic (C20:0)	0.46^b^	0.49^ab^	0.47^ab^	0.48^ab^	0.50^a^	0.005	0.054
Heneicosanoic (C21:0)	0.29	0.31	0.29	0.30	0.31	0.003	0.078
Behenic (C22:0)	0.53	0.56	0.54	0.54	0.58	0.006	0.069
Tricosanoic (C23:0)	1.24^b^	1.55^ab^	1.72^ab^	2.32^a^	2.19^a^	0.11	0.004
Lignoceric (C24:0)	0.51	0.54	0.51	0.52	0.36	0.03	0.252
MUFAs, g/100 g							
Myristoleic (C14:1)	0.12	0.13	0.13	0.13	0.13	0.002	0.0.193
Pentadecenoic (C15:1)	0.28	0.29	0.38	0.40	0.42	0.02	0.024
Palmitoleic (C16:1)	0.60	0.71	1.04	1.23	0.91	0.10	0.284
Heptadecenoic (C17:1)	0.20	0.21	0.21	0.21	0.22	0.002	0.128
Oleic (C18:1)	4.81	5.82	7.48	10.56	8.42	0.71	0.089
Gadoleic (C20:1)	0.30^b^	0.32^ab^	0.31^ab^	0.32^ab^	0.33^a^	0.004	0.017
PUFAs, g/100 g							
Linoleic (C18:2)	11.37^b^	12.86^ab^	14.84^ab^	27.41^a^	19.86^ab^	1.83	0.027
Alpha-Linolenic (C18:3)	0.31^b^	0.32^ab^	0.32^ab^	0.37^a^	0.35^ab^	0.007	0.016
Gamma-Linolenic (C18:3)	0.28^b^	0.29^ab^	0.28^ab^	0.31^ab^	0.31^a^	0.004	0.021
Eicosadienoic (C20:2)	0.35^c^	0.37^bc^	0.36^bc^	0.43^a^	0.41^ab^	0.007	0.001
Dihomo-γ-linolenic (C20:3)	0.67^b^	0.70^ab^	0.68^ab^	0.74^a^	0.73^ab^	0.009	0.021
Arachidonic (C20:4)	0.28	0.24	0.29	0.18	0.26	0.02	0.542
Eicosapentaenoic (C20:5)	0.29	0.31	0.30	0.30	0.32	0.004	0.072
Docosadienoic (C22:2)	0.28	0.30	0.29	0.29	0.31	0.003	0.078
Docosahexaenoic (C22:6)	0.26^b^	0.27^ab^	0.28^ab^	0.28^ab^	0.30^a^	0.004	0.009
ΣSFAs, g/100 g	15.45^b^	17.98^ab^	20.58^ab^	30.41^a^	25.03^ab^	1.70	0.033
ΣMUFAs, g/100 g	6.31	7.47	9.54	12.85	10.43	0.82	0.094
ΣPUFAs, g/100 g	14.09^b^	15.68^ab^	17.65^ab^	30.30^a^	22.87^ab^	1.85	0.028
∑n3, g/100 g	0.86^b^	0.91^ab^	0.89^ab^	0.95^a^	0.97^a^	0.01	0.007
∑n6, g/100 g	12.29^b^	13.76^ab^	15.78^ab^	28.32^a^	20.84^ab^	1.84	0.028

^a,b,c^Values in the same row with no common superscript differ significantly (*p* < 0.05).

SFAs: Saturated Fatty Acids; MUFAs: Monounsaturated Fatty Acids; PUFAs: Polyunsaturated Fatty Acids; n3: Omega-3 Polyunsaturated Fatty Acids: [Alpha-Linolenic (C18:3), Eicosapentaenoic (C20:5), Docosahexaenoic (C22:6)]; n6: Omega-6 Polyunsaturated Fatty Acids: [Linoleic (C18:2), Gamma-Linolenic (C18:3), Arachidonic (C20:4), Eicosadienoic (C20:2)].

The data in this table represent the actual measured content of fatty acids in the pectoralis major muscle across different experimental groups.

#### 3.4.2 Relative fatty acid content in pectoralis major muscle (%)

Table 7 shows that, compared to C group, the stearic acid (C18:0) content was significantly increased in 8% Sl (Full), 4% Sl (Phased), and 8% Sl (Phased) groups. The linoleic acid (C18:2) content in 4% Sl (Phased) group was significantly higher than that in 8% Sl (Full) group. Additionally, the total monounsaturated fatty acids (ΣMUFAs) in 4% Sl (Phased) group were significantly lower than in 8% Sl (Full) group, while the total polyunsaturated fatty acids (ΣPUFAs) and omega-6 fatty acid levels were significantly higher in 4% Sl (Phased) group compared to 8% Sl (Full) group (*p* < 0.05).

**Table 7:** Impact of Sl meal on breast meat fatty acid profile (relative percentages).

Item	C	4% Sl (Full)	8% Sl (Full)	4% Sl (Phased)	8% Sl (Phased)	SEM	*p*-value
SFAs, %							
Myristic (C14:0)	0.77	0.72	0.74	0.69	0.70	0.01	0.126
Pentadecanoic (C15:0)	0.35	0.35	0.32	0.19	0.28	0.02	0.110
Palmitic (C16:0)	24.32	24.57	24.36	25.17	24.39	0.26	0.862
Heptadecanoic (C17:0)	0.56	0.55	0.49	0.29	0.04	0.04	0.116
Stearic (C18:0)	8.25^c^	8.79^bc^	9.11^b^	9.33^ab^	9.96^a^	0.14	*p* < 0.001
Arachidic (C20:0)	1.36	1.32	1.19	0.67	1.04	0.09	0.128
Heneicosanoic (C21:0)	0.86	0.84	0.75	0.41	0.65	0.06	0.125
Behenic (C22:0)	1.58	1.54	1.38	0.76	1.20	0.11	0.125
Tricosanoic (C23:0)	3.52	3.97	3.95	3.20	4.06	0.17	0.444
Lignoceric (C24:0)	1.50	1.46	1.31	0.72	0.71	0.12	0.054
MUFAs, %							
Myristoleic (C14:1)	0.35	0.34	0.31	0.18	0.26	0.02	0.091
Pentadecenoic (C15:1)	0.79	0.82	0.95	0.57	0.84	0.07	0.548
Palmitoleic (C16:1)	1.67	1.64	1.97	1.63	1.50	0.07	0.234
Heptadecenoic (C17:1)	0.59	0.57	0.52	0.29	0.45	0.04	0.136
Oleic (C18:1)	13.24	13.97	15.17	14.11	14.05	0.24	0.172
Gadoleic (C20:1)	0.88	0.85	0.76	0.45	0.68	0.06	0.119
PUFAs, %							
Linoleic (C18:2)	30.80^ab^	29.54^ab^	29.14^b^	36.98^a^	32.26^ab^	0.94	0.048
Alpha-Linolenic (C18:3)	0.90	0.86	0.78	0.51	0.70	0.05	0.132
Gamma-Linolenic (C18:3)	0.83	0.79	0.72	0.43	0.63	0.05	0.132
Eicosadienoic (C20:2)	1.02	1.00	0.90	0.59	0.82	0.06	0.198
Dihomo-γ-linolenic (C20:3)	1.96	1.90	1.71	1.02	1.52	0.13	0.137
Arachidonic (C20:4)	0.84	0.62	0.74	0.26	0.46	0.07	0.073
Eicosapentaenoic (C20:5)	0.87	0.85	0.76	0.42	0.67	0.06	0.136
Docosadienoic (C22:2)	0.84	0.82	0.74	0.41	0.64	0.06	0.125
Docosahexaenoic (C22:6)	0.76	0.74	0.69	0.40	0.61	0.06	0.173
ΣSFAs, %	43.06	44.10	43.60	41.44	43.44	0.36	0.192
ΣMUFAs, %	17.51^ab^	18.19^ab^	19.69^a^	17.24^b^	17.79^ab^	0.26	0.017
ΣPUFAs, %	38.83^ab^	37.12^b^	36.18^b^	41.02^a^	38.31^ab^	0.48	0.011
∑n3, %	2.53	2.45	2.24	1.33	1.98	0.17	0.145
∑n6, %	34.43^ab^	32.85^ab^	32.31^b^	38.70^a^	34.87^ab^	0.73	0.043

^a,b,c^Values in the same row with no common superscript differ significantly (*p* < 0.05).

SFAs: Saturated Fatty Acids; MUFAs: Monounsaturated Fatty Acids; PUFAs: Polyunsaturated Fatty Acids; n3: Omega-3 Polyunsaturated Fatty Acids: [Alpha-Linolenic (C18:3), Eicosapentaenoic (C20:5), Docosahexaenoic (C22:6)]; n6: Omega-6 Polyunsaturated Fatty Acids: [Linoleic (C18:2), Gamma-Linolenic (C18:3), Arachidonic (C20:4), Eicosadienoic (C20:2)].

The data in this table represent the percentages of each fatty acid in the total amount of actually measured fatty acids.

### 3.5 Antioxidant capacity in the pectoralis major muscle

Table 8 shows that, the glutathione peroxidase level in 4% Sl (Phased) group was statistically significantly lower than that in 8% Sl (Full) group (*p* < 0.05).

**Table 8:** Impact of Sl meal on breast meat antioxidant capacity of broiler chickens.

Item	C	4% Sl (Full)	8% Sl (Full)	4% Sl (Phased)	8% Sl (Phased)	SEM	*p*-value
T-AOC, μmol/mL	0.60	0.54	0.67	0.67	0.55	0.02	0.096
CAT, μmol/min/mL	342.45	358.97	302.28	240.97	252.83	19.39	0.208
MDA, nmol/mL	1.50	3.08	3.05	3.36	2.66	0.26	0.191
SOD, U/mL	167.94	151.06	170.90	173.37	167.19	9.57	0.969
GSH-Px, nmol/min/mL	1,600.30^c^	2,356.95^ab^	2,465.88^a^	1,775.81^bc^	1,820.15^abc^	96.73	0.003

^a,b,c^Values in the same row with no common superscript differ significantly (*p* < 0.05).

### 3.6 Plasma immune and antioxidant biomarker analysis

Table 9 shows that, the levels of IFN-γ, IL-1β, IL-6, and TNF-α in the 4% Sl (Phased) and 8% Sl (Phased) groups were significantly higher than those in the C group (*p* < 0.05), while IFN-γ, IL-1β, and TNF-α levels were significantly lower than those in the 4% Sl (Full) and 8% Sl (Full) groups (*p* < 0.05). The IgA and IgG levels in the 4% Sl (Phased) and 8% Sl (Phased) groups were significantly higher than those in the C group (*p* < 0.05), and the IgM levels in the 4% Sl (Full), 8% Sl (Full), and 8% Sl (Phased) were significantly higher than those in the C and 4% Sl (Phased) groups (*p* < 0.05). The SOD activity in the 4% Sl (Full) and 4% Sl (Phased) groups was significantly higher than that in the 8% Sl (Phased) group (*p* < 0.05).

**Table 9:** Impact of Sl meal on plasma immunity and antioxidant capacity of broiler chickens.

Item	C	4% Sl (Full)	8% Sl (Full)	4% Sl (Phased)	8% Sl (Phased)	SEM	*p*-value
IFN-γ, pg/mL	41.52^d^	108.89^a^	96.39^b^	77.51^c^	74.39^c^	4.35	<0.001
IL-1β, pg/mL	71.97^d^	143.06^b^	169.13^a^	90.47^c^	101.97^c^	6.86	<0.001
IL-6, pg/mL	17.23^d^	31.60^b^	23.71^c^	25.34^c^	37.15^a^	1.33	<0.001
TNF-α, pg/mL	56.42^d^	102.76^a^	106.90^a^	68.09^c^	90.16^b^	3.78	<0.001
IgA, μg/mL	676.42^d^	1,201.78^b^	1,389.13^a^	929.12^c^	1,377.62^ab^	54.14	<0.001
IgG, μg/mL	12.92^c^	18.18^b^	20.11^b^	27.75^a^	18.61^b^	0.93	<0.001
IgM, μg/mL	2,050.95^c^	2,744.58^a^	2,403.42^b^	1,831.74^c^	2,864.20^a^	78.29	<0.001
T-AOC, μmol/mL	0.82	0.62	0.70	0.79	0.75	0.05	0.106
CAT, μmol/min/mL	38.45	35.15	37.01	34.25	30.90	1.34	0.482
MDA, nmol/mL	0.26^ab^	0.23^ab^	0.16^b^	0.26^ab^	0.37^a^	0.02	0.011
SOD, U/mL	21.58^ab^	24.12^a^	20.62^ab^	23.45^a^	15.63^b^	0.92	0.011
GSH-Px, nmol/min/mL	99.67	87.82	97.81	83.33	87.90	3.23	0.491

^a,b,c^Values in the same row with no common superscript differ significantly (*p* < 0.05).

### 3.7 Cecal microbial diversity

**Figure 1:**
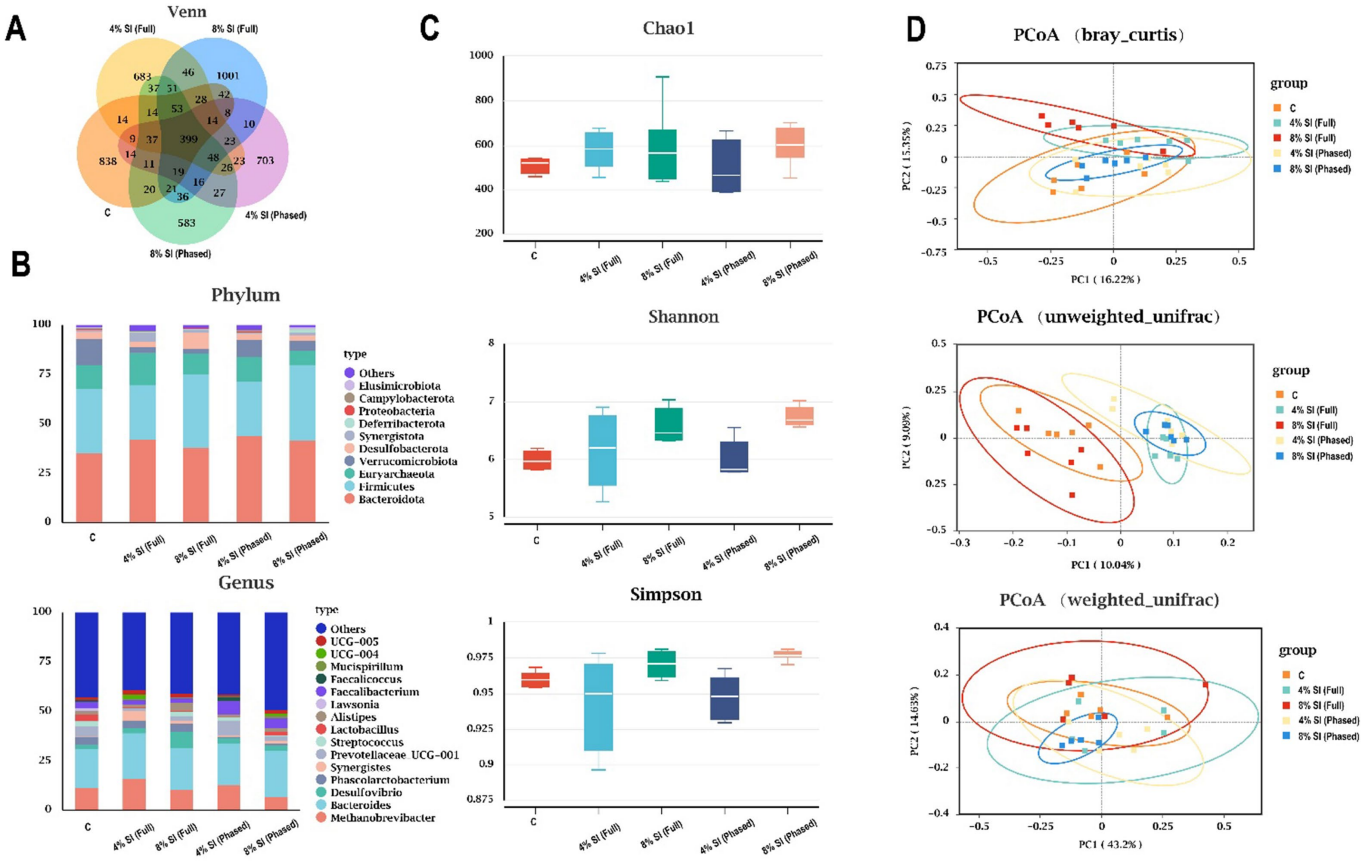
Impact of Sl meal on cecal microbiota composition and diversity of broiler chickens. **(A)** Venn diagram of core operational taxonomic units in the cecal chyme. **(B)** Relative abundance of cecal microbial community members at the phylum and genus level. **(C)** The α diversity parameters (Chao1, Shannon, Simpson) of cecal microbiota. **(D)** Principal coordinate analysis (PCoA) on cecal microbiota based on Bray-Curtis, weighted UniFrac, unweighted UniFrac. Asterisks (*, **, ***) denote statistical significance at *p < 0.05, **p < 0.01, and ***p < 0.001, respectively. “ns” indicates no significant difference.

Figure 1 shows that, venn diagram (Figure 1A) indicates 394 shared core OTUs across all groups, with unique OTUs identified in groups C, 4% Sl (Full), 8% Sl (Full), 4% Sl (Phased), and 8% Sl (Phased) groups numbering 838, 683, 1,001, 703, and 583, respectively. Figure 1B demonstrates that *Bacteroidota* and *Firmicutes* were the dominant phyla in all groups. Figure 1C reveals no significant differences in alpha diversity (Chao1, Simpson, and Shannon indices) among groups. Figure 1D, based on PCoA analysis (Bray-Curtis, weighted UniFrac, and unweighted UniFrac), shows distinct clustering of microbial composition across groups. Adonis tests further confirmed statistically significant beta diversity, indicating marked differences in microbial community structure among the groups.

**Figure 2:**
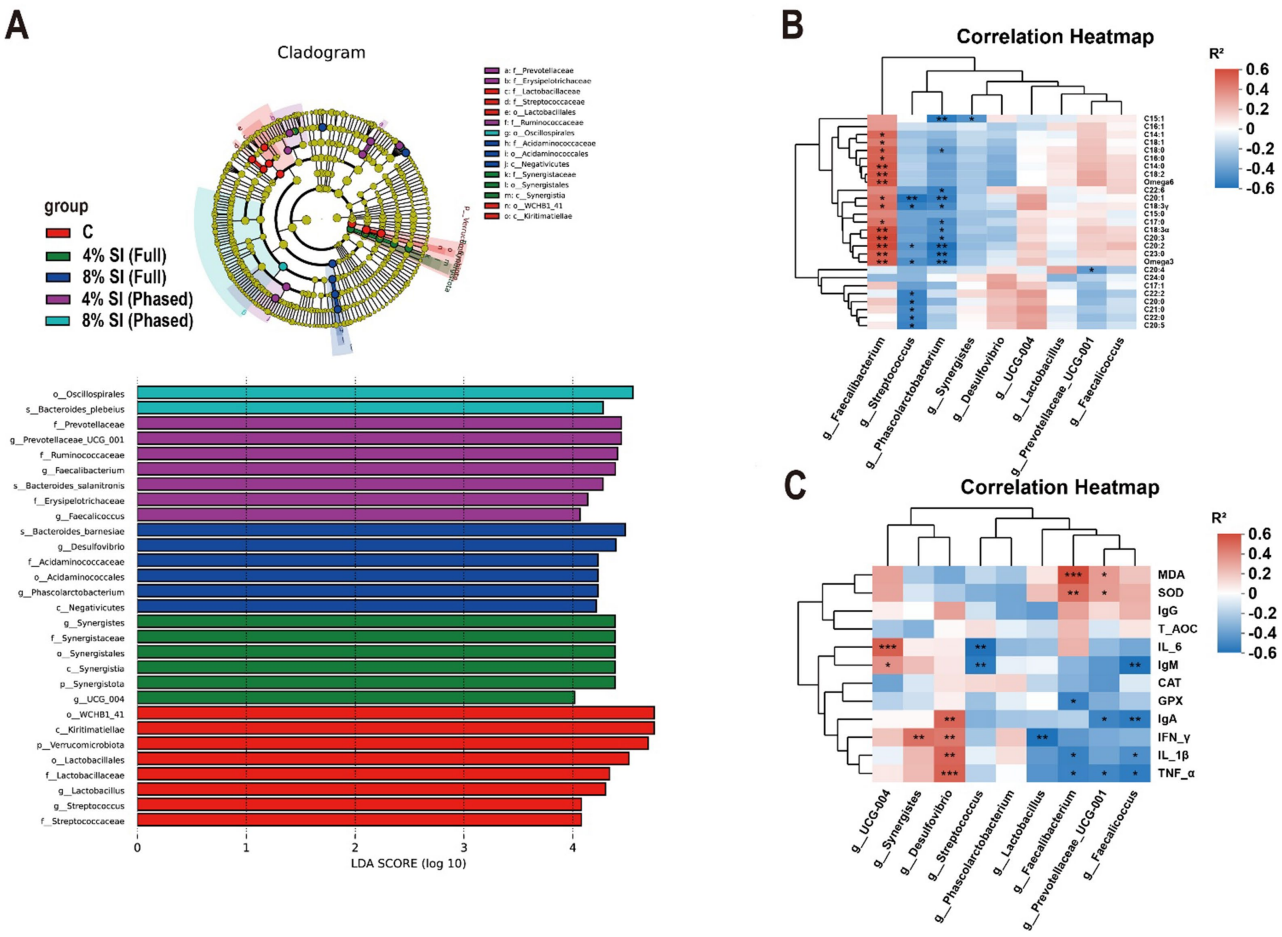
Association analysis of differentially abundant cecal microbiota with pectoral muscle fatty acids, plasma immune, and antioxidant parameters in yellow-feathered broilers. **(A)** Linear discriminant analysis (LDA) combined effect size measurements (LEfSe) analysis of cecal microbiota. **(B)** Correlation analysis between differentially abundant cecal taxa and host pectoral muscle fatty acid content. **(C)** Correlation analysis between differentially abundant cecal taxa and plasma immune and antioxidant parameters. Asterisks (*, **, ***) denote statistical significance at *p < 0.05, **p < 0.01, and ***p < 0.001, respectively. “ns” indicates no significant difference.

Figure 2 shows that, LEfSe analysis of cecal microbial taxa revealed distinct taxonomic enrichments across groups (Figure 2A). In C group, the genera *Lactobacillus* (g_Lactobacillus) and *Streptococcus* (g_Streptococcus) were significantly enriched. 4% Sl (Full) group exhibited enrichment of *Synergistes* (g_Synergistes) and *UCG_004* (g_UCG_004). 8% Sl (Full) group showed significant enrichment of *Desulfovibrio* (g_Desulfovibrio) and *Phascolarctobacterium* (g_Phascolarctobacterium), while 4% Sl (Phased) group displayed marked enrichment of *Prevotellaceae_UCG_001* (g_Prevotellaceae_UCG_001), *Faecalibacterium* (g_Faecalibacterium), and *Faecalicoccus* (g_Faecalicoccus).

Spearman correlation analysis further assessed associations between genus-level microbial differences and plasma antioxidant (Figure 2B)/immune (Figure 2C) indicators. In Figure 2C, enriched *Lactobacillus* correlated negatively with IFN-γ levels, while *Streptococcus* was inversely associated with IL-6 and IgM. In 4% Sl (Full) group, *Synergistes* positively correlated with IFN-γ, and *UCG_004* showed positive links to IL-6 and IgM. 8% Sl (Full) group-enriched *Desulfovibrio* exhibited positive correlations with IgA, IFN-γ, IL-1β, and TNF-α. For 4% Sl (Phased) group, *Prevotellaceae_UCG_001* positively correlated with MDA and SOD but negatively with IgA and TNF-α; *Faecalibacterium* was positively linked to MDA and SOD yet inversely related to GSH-Px, IL-1β, and TNF-α; *Faecalicoccus* showed negative associations with IgM, IgA, IL-1β, and TNF-α.

## 4 Discussion

The efficacy of insect-based feed ingredients in animal diets is highly dependent on their inclusion levels and animal growth stages, as evidenced by dose- and stage-dependent responses across species. Excessive supplementation (e.g., 36–40% BSF meal in fish (17) or complete soybean meal replacement in piglets (20)) consistently impaired growth performance, whereas moderate doses sustained productivity. Notably, adverse effects often manifest during specific physiological phases, such as the latter stages of Rhode Island Red × Fayoumi crossbred chickens (19) or piglet development. Given that 42–63 days represents the peak absolute growth rate phase in fast-growing yellow-feathered broilers, this study strategically targeted this period as the *Scarabaeiform larva* (Sl) meal withdrawal window to contrast full-term (1–63 days) versus early-mid phased (1–42 days) supplementation regimes.

Growth performance: During the 1–42 days period, 4% Sl (Full) and 4% Sl (Phased) groups (4% Sl meal supplementation) exhibited significantly higher body weight, weight gain, and lower FCR compared to the control, 8% Sl (Full), and 8% Sl (Phased) groups. From days 43–63, 4% Sl (Phased) and 8% Sl (Phased) groups (Sl meal withdrawn) exhibited significantly higher weight gain than the continuously supplemented 4% Sl (Full) and 8% Sl (Full) groups, with 4% Sl (Phased) group achieving the highest final body weight among all groups. These results indicate that low-dose Sl meal supplementation (4%) during the early-mid growth phase (1–42 days) improves broiler growth performance, while continued supplementation in later stages (43–63 days) negatively affects growth. This result aligns with previous studies indicating that insect meal supplementation at appropriate levels promotes animal growth performance, whereas excessive use impairs growth (30, 31). The negative effects may be associated with the long-term accumulation of anti-nutritional factors such as chitin and enzyme inhibitors (e.g., phenols or tannins) present in insect meal (32). Chitin, a natural polysaccharide ubiquitously present in insect exoskeletons, is difficult to decompose by digestive enzymes in the animal gut. It can bind with nutrients like proteins and minerals to form complexes, thereby reducing their digestibility and absorption rate (20). Phenols and tannins can bind to digestive enzymes (e.g., proteases, amylases), inhibiting their activity (33). The optimal growth performance observed in the 4% Sl (Phased) group in this trial may be attributed to the reduced accumulation of insect-derived anti-nutritional factors resulting from the lower dosage and phased supplementation strategy.

Slaughter performance and meat quality: The increased abdominal fat ratio in 4% Sl (Phased) and 8% Sl (Phased) groups suggests that early-mid supplementation with Sl meal may promote lipid deposition in yellow-feathered broilers. As a key determinant of meat flavor, fat significantly influences sensory attributes and palatability (34, 35). Meat quality analysis of breast muscle revealed higher absolute intramuscular fat content in 4% Sl (Phased) and 8% Sl (Phased) groups compared to the control. Further analysis of fatty acid composition demonstrated significant increases in absolute levels of multiple fatty acids in 4% Sl (Phased) and 8% Sl (Phased) groups, including myristic acid (C14:0), α-linolenic acid (C18:3), palmitic acid (C16:0), and linoleic acid (C18:2). Elevated absolute saturated fatty acids (SFAs) such as myristic and palmitic acids may enhance meat texture by increasing fat melting point and hardness (36), while higher absolute polyunsaturated fatty acids (PUFAs) like linoleic and α-linolenic acids could generate plant-derived aldehydes (e.g., hexanal) and 2-pentylfuran through lipid oxidation, improving meat aroma (37–39).

The absolute levels of omega-3 fatty acids (e.g., α-linolenic acid, docosahexaenoic acid) increased in 4% Sl (Phased) and 8% Sl (Phased) groups. This may enhance cardiovascular benefits (40). However, the elevated absolute omega-6 fatty acids in 4% Sl (Phased) group might promote immune hyperactivity, increasing risks of autoimmune disorders (41). Notably, the relative proportions of omega-6 to omega-3 fatty acids remained unchanged, indicating that the observed increases were driven by higher total lipid content rather than compositional shifts. Additionally, 4% Sl (Phased) group exhibited significantly higher absolute ΣPUFAs content than C. High PUFAs levels are prone to free radical attack, accelerating lipid oxidation and reducing shelf life. Antioxidant capacity assays showed no significant differences in breast muscle antioxidant activity (e.g., T-AOC, SOD) between 4% Sl (Phased)/8% Sl (Phased) groups and C group. However, 4% Sl (Full) and 8% Sl (Full) groups displayed significantly higher glutathione peroxidase (GSH-Px) activity than C group, suggesting that Sl meal supplementation enhances antioxidant capacity. The reduced GSH-Px in 4% Sl (Phased)/8% Sl (Phased) groups may result from PUFAs-induced oxidative consumption or the loss of Sl-derived antioxidant benefits after supplementation withdrawal.

Meat color analysis revealed significantly higher lightness (L*) in Sl-supplemented groups (4% Sl (Full), 8% Sl (Full), 4% Sl (Phased), 8% Sl (Phased)) compared to C group, with 4% Sl (Phased) group showing lower redness (a*) than 4% Sl (Full) group. This phenomenon may be attributed to reduced light scattering caused by uniform intramuscular fat distribution (42).

Immune response: The use of insect meal can significantly impact the immune performance of animals. Compared to the control group (C), 4% Sl (Full) and 8% Sl (Full) groups exhibited significantly elevated levels of pro-inflammatory cytokines (IFN-γ, IL-1β, IL-6, TNF-α), indicating that full-term supplementation with Sl meal may induce potential inflammatory responses in yellow-feathered broilers. Although cytokine levels in 4% Sl (Phased) and 8% Sl (Phased) groups remained higher than in C group, they were significantly lower than in 4% Sl (Full) and 8% Sl (Full) groups, suggesting that discontinuing Sl supplementation in later stages alleviates its pro-inflammatory effects. The preserved growth performance of 4% Sl (Phased) and 8% Sl (Phased) groups during the late phase implies that excessive immune activation triggered by upregulated cytokines likely contributed to the growth impairment observed in 4% Sl (Full) and 8% Sl (Full) groups (43).

Immunoglobulins are core indicators of humoral immune function (44). Immunoglobulin G (IgG), the primary contributor to humoral immunity (45), and immunoglobulin A (IgA), which maintains intestinal homeostasis and protects against bacterial infections (46), were significantly higher in 4% Sl (Phased) and 8% Sl (Phased) groups compared to C, indicating enhanced mucosal and systemic immunity. In contrast, 4% Sl (Phased) group had lower IgM levels than 4% Sl (Full), 8% Sl (Full), and 8% Sl (Phased) groups. As IgM is the first antibody produced during primary immune responses (47), this finding, combined with attenuated pro-inflammatory cytokine profiles, demonstrates that 4% Sl (Phased) and 8% Sl (Phased) groups achieved balanced immune activation-maintaining protective immunoglobulin levels while avoiding the hyperimmune state seen in 4% Sl (Full)/8% Sl (Full) groups. The immune marker trends align with previous studies on *Tenebrio molitor* and *Hermetia illucens* proteins in broilers, confirming that excessive insect meal supplementation induces immune hyperactivation and impairs growth performance (48).

Antioxidant capacity is critical as oxidative stress generates excessive free radicals that damage cells, DNA, and proteins, compromising physiological functions (49). Superoxide dismutase (SOD), a key antioxidant enzyme, catalyzes the conversion of superoxide radicals to hydrogen peroxide, which is subsequently detoxified to water by glutathione peroxidase (GSH-Px) and catalase (50). 4% Sl (Full) and 4% Sl (Phased) groups showed significantly higher SOD activity than 8% Sl (Phased) group, indicating that early 4% Sl supplementation enhances systemic antioxidant capacity. The sustained antioxidant activity in 4% Sl (Phased) group after Sl withdrawal suggests residual benefits, whereas excessive Sl use (8% Sl (Phased) group) may compromise redox homeostasis.

The gut microbiota maintains a symbiotic relationship with the host, and compositional shifts in its community can influence host metabolism and immune function (51, 52). This mechanistic link is further validated by fecal microbiota transplantation studies: for instance, transplantation of intestinal microbiota from high-growth-performance chickens into 1-day-old chicks significantly improved growth performance and intestinal development in the recipient chickens (53); similarly, transplantation of gut microbiota from mastitis cows into mice successfully induced mastitis in the mice (54). These cases underscore the critical impact of intestinal microbiota homeostasis on host growth and systemic immunity. In this trial, while species richness and evenness remained relatively stable across groups, microbial community structures differed significantly. Correlation analysis between group-specific bacterial taxa and host immune markers/fatty acid profiles revealed that *Streptococcus* (enriched in the control group, C) negatively correlated with multiple fatty acids, suggesting its metabolic activity or niche occupation may inhibit fatty acid synthesis/accumulation or promote catabolism in breast muscle. In contrast, *Faecalibacterium* (enriched in 4% Sl (Phased) group) positively correlated with fatty acids such as myristic acid (C14:0) and linoleic acid (C18:2). Previous studies indicate that *Faecalibacterium* can metabolically produce butyrate and enhances intestinal barrier function by promoting tight junction protein expression (55–57), alleviates diarrhea in young animals, and is considered a potential candidate for probiotic development (58). Moreover, butyrate, a metabolite of Faecalibacterium, serves as a crucial energy source that promotes lipid deposition in the body, which might be one reason for the higher feed utilization efficiency and improved breast muscle fatty acid composition in the 4% Sl (Phased) group (59). Notably, *Faecalibacterium* exhibited positive correlations with malondialdehyde (MDA) and superoxide dismutase (SOD) but negative correlations with glutathione peroxidase (GPX), IL-1β, and TNF-α, indicating its dual influence on oxidative stress modulation and inflammatory suppression. Furthermore, *Faecalicoccus* (enriched in 4% Sl (Phased) group) negatively correlated with IgM, IgA, IL-1β, and TNF-α, reinforcing the microbiota-driven immune optimization in 4% Sl (Phased) group. In 4% Sl (Full) group, *Synergistes* positively correlated with IFN-γ, while *UCG_004* associated with IL-6 and IgM. In 8% Sl (Full) group, *Desulfovibrio* positively correlated with IgA, IFN-γ, IL-1β, and TNF-α. These findings demonstrate that specific microbial taxa critically regulate immune factor expression, highlighting the gut microbiota’s systemic impact on both local intestinal and systemic immunity as well as lipid metabolism.

### 4.1 Limitations

This trial demonstrated the effects of phased feeding with Sl meal on broiler growth performance, lipid deposition, immune function, and gut microbiota. However, the absence of histopathological analysis using tissue sections represents a limitation. Furthermore, the active components of Sl meal (e.g., chitin, enzyme inhibitors, antimicrobial peptides, or other constituents) and their mechanisms of action remain unelucidated. Although beneficial effects of phased supplementation were observed, the phased data lack comprehensiveness. Specifically, optimal application stages and optimal dosage (around 4% inclusion level) warrant further refinement. Moreover, the economic feasibility of Sl meal production for feeding has not been investigated. Further studies are required to isolate and characterize these components, clarify the mechanisms underlying their adverse effects, optimize phased feeding strategies, conduct economic feasibility analysis, and ultimately advance scalable applications of Sl meal in poultry feed production.

## 5 Conclusion

This study demonstrates that a phased feeding strategy incorporating 4% Sl meal into the diet during the early-mid growth phase (1–42 days) and transitioning to Sl-free diets in the later phase (43–63 days) significantly enhances growth performance and final body weight in yellow-feathered broilers. This strategy also improves carcass quality (promoting lipid deposition, improving breast meat quality), boosts immune function, and modulates gut microbiota. Critically, this approach effectively avoids the growth inhibition problems caused by full-term or excessive Sl supplementation. Therefore, we recommend implementing this phased Sl feeding strategy in practical broiler production to maximize benefits while mitigating risks. Future research should focus on: (1) Isolating and identifying the key bioactive compounds in Sl meal responsible for its primary effects, investigating and elucidating their mechanisms of action, and mitigating the potential adverse effects of Sl meal; and (2) Conducting rigorous cost–benefit analyses to evaluate its economic viability and practical applicability under commercial production conditions.

## Data Availability

The data presented in this study are deposited in the NCBI BioProject repository under accession number PRJNA1287074.
