# Supplementary material for: Changes in growth performance, immune function, and meat quality of yellow-feathered broilers fed Scarabaeiform larvae meal during early-mid growth phases
Source: Front Vet Sci. 2025 Aug 8;12:1638495. doi: 10.3389/fvets.2025.1638495 (PMC12370514; doi:10.3389/fvets.2025.1638495)
Supplement: Supplementary file 1 [file Table_1.docx]

Supplementary Material

# Supplementary Tables

**Table1** PERMANOVA (Adonis) results for beta-diversity analysis of microbial communities

| Distance algorithm | F.Model | R^2^ | *P*-value |
| --- | --- | --- | --- |
| bray_curtis | 3.476 | 0.357 | 0.001 |
| weighted_unifrac | 2.221 | 0.262 | 0.006 |
| unweighted_unifrac | 1.954 | 0.238 | 0.001 |
